# Prognostic Impact of Para-Aortic Lymph Node Metastasis in Resected Non-Pancreatic Periampullary Cancers

**DOI:** 10.1245/s10434-024-15847-z

**Published:** 2024-07-20

**Authors:** Kaival Gundavda, Amit Chopde, Avinash Pujari, Bhaskar Reddy, Akash Pawar, Anant Ramaswamy, Vikas Ostwal, Shraddha Patkar, Manish Bhandare, Shailesh V. Shrikhande, Vikram A. Chaudhari

**Affiliations:** 1 Division of Gastrointestinal and HPB Surgery, Department of Surgical Oncology, Tata Memorial Hospital, Homi Bhabha National Institute (HBNI), Mumbai, Maharashtra India; 2 Department of Biostatistics, Tata Memorial Hospital, Homi Bhabha National Institute (HBNI), Mumbai, Maharashtra India; 3 Department of Medical Oncology, Tata Memorial Hospital, Homi Bhabha National Institute (HBNI), Mumbai, Maharashtra India

**Keywords:** Para-aortic lymph node, Station 16b1, Non-pancreatic periampullary cancer, Resectable periampullary cancer, PALN, Pancreaticoduodenectomy

## Abstract

**Background:**

Surgery remains debatable in para-aortic lymph node (PALN, station 16b1) metastasis in non-pancreatic periampullary cancer (NPPAC). This study examined the impact of PALN metastasis on outcomes following pancreaticoduodenectomy (PD) in NPPAC.

**Methods:**

A retrospective analysis of patients with NPPAC who were explored for PD with PALN dissection was performed. Based on the extent of nodal involvement on final histopathology, they were stratified as node-negative (N0), regional node involved (N+) and metastatic PALN (N16+) and their outcomes were compared.

**Results:**

Between 2011 and 2022, 153/887 PD patients underwent a PALN dissection, revealing N16+ in 42 patients (27.4%), of whom 32 patients underwent resection. The 3-years overall survival (OS) for patients with N16+ was 28% (95% confidence interval [CI] 13–60%), notably lower than the 67% (95% CI 53–83.5%; *p* = 0.007) for those without PALN metastasis. Stratified by nodal involvement, the median OS for N+ and N16+ patients was similar (28.4 months and 26.2 months, respectively). The N0 subgroup had a significantly longer 3-years OS of 87.5% (95% CI 79–96.7%; *p* = 0.0051). Interestingly, 10 patients not offered resection following N16+ identified on frozen section had a median survival of only 9 months. The perioperative morbidity and mortality in patients undergoing PD with PALN dissection were similar to standard resections.

**Conclusion:**

In a select group of patients with NPPAC, PD in isolated PALN metastasis was associated with improved OS. The survival in this group of patients was comparable with regional node-positive patients and significantly better than palliative treatment alone.

**Supplementary material:**

The online version of this article (10.1245/s10434-024-15847-z) contains supplementary material, which is available to authorized users.

Pancreaticoduodenectomy (PD) is the standard treatment for resectable periampullary cancers. Standard lymphadenectomy for PD includes resection of nodes along the hepatoduodenal ligament (stations 5, 6, 8a, 12b1, 12b2, 12c), the retropancreatic nodes (station 13a, 13b), nodes along the superior mesenteric artery (station 14a, 14b), and anterior surface of the pancreatic head (station 17a, and 17b).^1^ Lymph node status is an important predictor of prognosis, however the role of resection in the setting of positive para-aortic lymph nodes (PALN) for periampullary cancers is not well-defined.^1^

PALN (station 16b1) metastasis in pancreatic ductal adenocarcinoma (PDAC) portends a poor prognosis and is associated with reduced survival.^2–4^ The recommendations for the treatment of PDAC with metastatic para-aortic nodes remain varied. Some recommend abandoning curative resections, while others treat it as an isolated metastatic disease, where potential curative surgery is feasible in combination with systemic therapy.^3–9^ Similarly, the significance of PALN metastasis and the role of radical resection in non-pancreatic periampullary cancer (NPPAC) also remains contentious. In the past two decades, only a few retrospective studies have sought to answer this question.^10–15^ Matched for stage, the prognosis for NPPAC is better as compared with PDAC.^16^ Radical resection in PALN-positive patients may provide survival benefit in NPPAC in contrast to PDAC.

PD is a complex surgical procedure and is inherently associated with significant morbidity.^16^ The potential survival benefit of a radical resection in advanced disease therefore needs to justify the morbidity and potential mortality risk associated with surgery. Over the last two decades, high-volume centers across the world have consistently reported improved perioperative outcomes for PD. Although the major morbidity rate remains around 30%, mortality rates of around 2–3% have consistently been reported as compared with historical figures of more than 5%.^16,17^

We hypothesized that in patients with NPPAC with limited PALN involvement as a solitary site of metastasis, radial resection might be associated with better survival as compared with chemotherapy alone, provided a margin-negative resection is achieved, without a significant increase in morbidity. This study aimed to contribute to the understanding of the role of radical resection in the treatment of NPPAC with isolated PALN involvement, with a focus on survival outcomes and its implications for clinical decision making and treatment strategies.

## Materials and Methods

The study included patients with suspected or biopsy-proven non-pancreatic periampullary adenocarcinoma who underwent PD in the Gastro-Intestinal Disease Management Group at Tata Memorial Centre, Mumbai, India, between 2011 and 2022.

The preoperative evaluation included contrast-enhanced cross-sectional imaging and a serum carcinoembryonic antigen (CEA) and carbohydrate antigen (CA)-19.9 level estimation. The preferred initial imaging modality was a triphasic contrast-enhanced computed tomography scan of the thorax, abdomen, and pelvis (CECT TAP) with a pancreatic protocol.^18^ Magnetic resonance imaging (MRI)/magnetic resonance cholangiopancreatography (MRCP) or 18-fluoro-deoxyglucose positron emission tomography (FDG-PET) were used selectively. A side-viewing endoscopy (SVE) and/or endoscopic ultrasound (EUS) were performed in select cases whenever indicated to document the lesion and to obtain tissue diagnosis. In patients who had undergone a biopsy elsewhere, a pathology review was obtained at our institute.

All patients with suspected periampullary cancers (arising from the ampulla of Vater, distal common bile duct [CBD], and duodenum), thought to be resectable on imaging, were discussed in a multidisciplinary joint clinic and then planned for surgical exploration. Cancers that originated from the pancreatic head and those with non-adenocarcinoma histology were excluded from the analysis.

Preoperative biliary drainage was performed in patients with elevated bilirubin >15 mg/dL, features of cholangitis, or in patients needing presurgery rehabilitation or neoadjuvant therapy. Surgical resection was planned after 4–6 weeks of biliary drainage.^19^

All patients underwent a PD with a standard lymphadenectomy.^1,16^ Resection was committed after confirming the absence of gross metastatic disease. A Kocher maneuver was performed and PALN (station 16b1) dissection (sampling or clearance) was carried out in patients with radiologically indeterminate or suspicious PALN, or an intraoperative suspicion of nodal involvement. The PALN ‘sampling’ involved a limited excision of the fibro-fatty tissue in the inter-aortocaval region (16b1 int) between the lower border of the left renal vein and the origin of the inferior mesenteric artery (IMA), along with the left PALNs bounded laterally by the left gonadal vein (16b1 lat). A systematic ‘clearance’ of this template was performed if a frozen section (FS) analysis confirmed metastasis.

The International Study Group of Pancreatic Surgery (ISGPS) definitions were used to define complications such as postoperative pancreatic fistula (POPF), post-pancreatectomy hemorrhage (PPH) and delayed gastric emptying (DGE). POPF B and POPF C together were considered CR-POPF as per the 2016 ISGPS consensus guidelines.^20–23^ Postoperative complications were recorded following the Clavien–Dindo classification system, with grades IIIa and above being considered significant morbidity.^24^ Deaths occurring within 90 days of surgery were considered as postoperative mortality.

Patients were categorized into three groups as per the extent of nodal disease on final histopathology: (1) no para-aortic or regional node metastasis (PALN −ve, regional node −ve) = [N0]; (2) no para-aortic metastasis but regional nodal involvement (PALN −ve, regional node +ve) = [N+]; (3) resection in isolated metastatic para-aortic nodes (PD with PALN+) = [N16+].

The demographic, histopathological, and outcome variables were compared between the groups. Pathology review included assessment of tumor epicenter, tumor size, histological differentiation, lymphovascular invasion (LVI) and perineural invasion (PNI). In deeply infiltrating lesions causing architectural distortion, the epicenter characterization into ampullary, distal bile duct or duodenum is sometimes uncertain. These NPPAC were classified as periampullary tumors not otherwise specified (NOS).

Adjuvant chemotherapy was administered in fit patients with T3/T4 and/or node-positive disease. Adjuvant radiation therapy (RT) was considered selectively in margin-positive resections or extensive nodal metastasis after a multidisciplinary discussion.

The data of the present study were collected in the course of common clinical practice and, accordingly, the signed informed consent was obtained from each patient for any surgical and clinical procedure. The study protocol was in accordance with the ethical standards of the Institutional Research Committee and the 1964 Helsinki Declaration and its later amendments.

### Statistics

Outcome variables included complication rate, recurrence, and overall survival (OS) for the above-mentioned groups. OS was calculated from the date of diagnosis to the date of death or the last follow-up, while disease-free survival (DFS) was calculated from the date of surgery to the date of clinical or radiological evidence of disease recurrence. Survival estimation was performed using the Kaplan–Meier survival function, and the Cox proportional hazards model was used for multivariate analysis to determine the significance of variables found to be significant in univariate analysis. All analysis was performed using Statistical Product and Service Solutions (SPSS) version 26 (IBM Corporation, Armonk, NY, USA) and a *p*-value <0.05 was considered statistically significant.

## Results

### Patient Cohort, and Operative and Histopathological Characteristics

Overall, 887 patients with suspected or biopsy-proven NPPAC underwent PD in the study period, of whom 153 patients (17.2%) underwent PD with PALN sampling.

The median age of the patients was 56 years (range 32–82 years), with a 60% male predominance. On clinico-radiologic evaluation, the tumors were most commonly epicentered at the ampulla (78.3%), followed by the distal CBD (18.2%) and the duodenum (3.5%). Seven of the 153 resected patients received chemotherapy with neoadjuvant intent. Three patients had suspicious nodal disease on imaging, three had an elevated CA-19.9, and one patient received chemotherapy before presentation at our institute (Table 1).

**Table 1:** Patient demographics and operative characteristics

		PALN+	PALN− [*n* = 111]		
		N16+ [*n* = 32]	N+ [*n* = 52]	N0 [*n* = 59]	*p*-Value
Mean age in years (Standard Deviation)		52.84 (11.049)	57.55 (10.896)	55.915 (11.422)	0.174
Sex	Male	17 (53.1)	31 (59.6)	38 (64.4)	0.574
	Female	15 (46.9)	21 (40.4)	21 (35.6)	
Clinico-radiological primary site	Ampulla	26 (75)	40 (80)	46 (78.0)	0.478
	CBD	8 (25)	9 (18)	9 (15.5)	
	Duodenum	0 (0.0)	1 (2)	4 (6.8)	
Preoperative biliary drainage	None	6 (18.8)	10 (19.2)	8 (13.6)	0.727
	ERCP	23 (71.9)	37 (71.2)	45 (76.3)	
	PTBD	0 (0.0)	3 (5.8)	3 (5.1)	
	Others	3 (9.4)	2 (3.8)	3 (5.1)	
Neoadjuvant chemotherapy	Yes	0 (0.0)	4 (7.7)	3 (5.1)	0.283
	No	32 (100.0)	48 (92.3)	56 (94.9)	
Surgery approach	Open	31 (96.9)	46 (88.5)	55 (93.2)	0.351
	Robotic	1 (3.1)	6 (11.5)	4 (6.8)	
Vascular resection	Yes	2 (6.2)	3 (5.8)	0 (0.0)	0.161
	No	30 (93.8)	49 (94.2)	59 (100.0)	
Adjuvant therapy	Observation	0 (0.0)	3 (5.8)	29 (49.2)	**< 0.05**
	CT	22 (68.8)	48 (92.3)	23 (39.0)	
	CTRT	5 (15.6)	0 (0.0)	1 (1.7)	
	NA	5 (15.6)	1 (1.9)	6 (10.2)	

Bold value indicates significant *p*-value

*PALN* para-aortic lymph node, *CBD* common bile duct, *ERCP* endoscopic retrograde cholangiopancreatography, *PTBD* percutaneous transhepatic biliary drainage, *CT* chemotherapy, *CTRT* concurrent chemoradiotherapy, *NA* not available

Data are expressed as *n* (%) unless otherwise specified

The baseline clinical, demographic, and operative characteristics are elaborated in Table 1. All patients underwent a PD. Pylorus-preserving PD was performed in 70.6% (101/143) of patients, while 37 underwent a classical Whipple’s procedure (25.8%).

Among the 153 patients where PALN sampling or dissection was performed, the decision was prompted by either the presence of radiological indeterminate PALN (*n* = 31, 3.5%), or an intraoperative suspicion of involved PALN or locally advanced disease (*n* = 82, 9.24%). In 40 patients (4.5%), sampling was performed at the surgeon’s discretion.

**Fig. 1:**
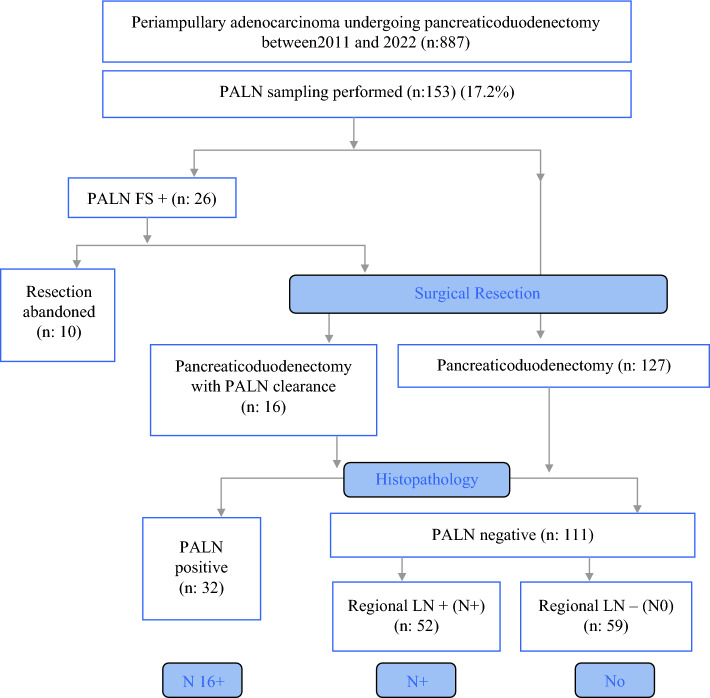
Evaluation and treatment algorithm for non-pancreatic periampullary cancers undergoing exploration with PALN sampling. *PALN* para-aortic lymph node, *FS* frozen section, *LN* lymph node

A FS analysis of PALN was performed in 86 patients (56.2%), of whom 26 patients had metastasis to PALN (PALN FS+). Among these 26 patients, PD with station 16b1 clearance was performed in 16 patients, while resection was abandoned in 10. The decision to abandon curative resection in these 10 cases was made based on careful consideration of advanced age, medical comorbidities in a ‘high-risk’ pancreas, and/or extensive retroperitoneal nodal disease not amenable for R0 resection. Five patients had multiple station PALN involvement, three patients had multiple comorbidities (two with coronary artery disease and one with hepatic cirrhosis), and two patients were >70 years of age with borderline performance status. These cases were treated with chemotherapy alone. These cases were analyzed separately and survival was compared with the resected N16+ patients (Fig. 1).

Among the 67 patients (43.8%) for whom FS analysis of the sampled PALN was not performed, 16 (23.8%) were found to have metastasis on final histopathology. Finally, a total of 42 patients (27.4%) were found to have metastasis to PALN on histopathology (N16+). Of the 111 patients with negative PALN, 52 were found to have regional node-positive disease (N+), whereas 59 were node-negative (N0) (Fig. 1).

There was no significant difference in the clinical and surgical characteristics between N16+, N+, and N0 patients. The three subgroups were similar in terms of sex (*p* = 0.574), median age (*p* = 0.174), clinico-radiological site of primary (*p* = 0.478), and preoperative biliary drainage (*p* = 0.727) (Table 1).

In the N16+ group, a median of four PALNs were sampled (range 1–19 nodes). A median of two lymph nodes were reported positive (range 1–6 nodes). All 32 N16+ cases who underwent PD were associated with regional lymph node metastasis; over 80% of these had pN2 disease (*p* < 0.05). The median regional node harvest was 26 nodes (12–55 nodes) with a median of seven positive regional nodes (range 2–30 nodes).

A significant correlation between increasing tumor size (*p* = 0.045), pT stage, and nodal status (pN) was identified (*p* < 0.05). As compared with N0, N+ and N16+ patients were more frequently associated with T3/T4 tumors and larger tumors (*p* < 0.05). A significant correlation between the presence of LVI and PNI was also observed (*p* < 0.05). The histopathological characteristics are depicted in Table 2.

**Table 2:** Histopathological characteristics

		PALN+	PALN− [*n* = 111]		*p*-value
		N16+ [*n* = 32]	N+ [*n* = 52]	N0 [*n* = 59]	
Tumor differentiation	Moderately differentiated	27 (84.4)	40 (76.9)	45 (76.3)	**0.033**
	Well differentiated	0 (0.0)	0 (0.0)	6 (10.2)	
	Poorly differentiated	4 (12.5)	12 (23.1)	6 (10.2)	
	NA	1 (3.1)	0 (0.0)	2 (3.4)	
Median tumor size [range (IQR)]		2.5 (2, 3.5)	2.25 (1.8, 3.5)	2 (1.5, 2.5)	**0.045**
Median LN harvest [range (IQR)]		26.5 (18, 33.75)	28.5 (21, 35.75)	23 (17, 32)	0.052
LN ratio [median (IQR)]		0.34 (0.19, 0.42)	0.86 (0.05, 0.162)	0	**< 0.05**
PALN resected [median (IQR)]		4 (3, 6.75)	3 (1.25, 5)	3 (2, 5)	0.152
LVI	Present	18 (56.2)	27 (51.9)	9 (15.3)	**< 0.05**
	Absent	14 (43.8)	25 (48.1)	50 (84.7)	
PNI	Present	18 (56.2)	27 (51.9)	9 (15.3)	**< 0.05**
	Absent	14 (43.8)	25 (48.1)	50 (84.7)	
Margins	Free	27 (84.4)	49 (94.2)	54 (91.5)	0.305
	Involved	5 (15.6)	3 (5.8)	5 (8.5)	
HPR tumor site	Ampulla	3 (9.3)	14 (26.9)	16 (27.1)	**0.02**
	Distal CBD	7 (21.9)	9 (17.3)	8 (13.6)	
	Periampullary, NOS	22 (68.8)	27 (51.9)	28 (47.5)	
	Duodenum	0 (0.0)	2 (3.8)	7 (11.9)	
Ampullary subtype	Intestinal	2 (6.5)	8 (15.4)	14 (25.9)	0.065
	Pancreatobiliary	6 (19.4)	21 (40.4)	13 (24.1)	
	Mixed	2 (6.5)	2 (3.8)	5 (9.3)	
	Not mentioned	20 (64.5)	20 (38.5)	19 (35.2)	
	Biliary type	1 (3.1)	1 (1.9)	3 (5.6)	
pT stage	T1	0 (0)	4 (7.7)	18 (31.6)	**< 0.05**
	T2	8 (25)	21 (40.4)	25 (43.9)	
	T3	21 (65.6)	27 (51.9)	13 (22.8)	
	T4	3 (9.4)	0 (0)	1 (1.8)	
pN stage	N0	0 (0.0)	0 (0.0)	59 (100.0)	**< 0.05**
	N1	5 (15.6)	33 (63.5)	0 (0.0)	
	N2	27 (84.4)	19 (36.5)	0 (0.0)	

Bold values indicate significant *p*-value

*PALN* para-aortic lymph node, *IQR* interquartile range, *LVI* lymphovascular invasion, *PNI* perineural invasion, *HPR* histopathology report, *LN* lymph node, *NA* not available, *NOS* not otherwise specified, *CBD* common bile duct

Data are expressed as *n* (%) unless otherwise specified

### Morbidity and Mortality Comparison

The major morbidity rate (Clavien–Dindo grades IIIa and above) was 38% (55/143 patients) and the postoperative mortality rate was 4.9% (7/143 patients). Four patients had multiorgan dysfunction and sepsis, secondary to POPF-C (two patients), PPH-C (one patient), and postoperative acute pancreatitis (POAP; one patient). Three patients suffered cardiac events in the postoperative period. Performance of PALN dissection in addition to standard PD did not increase postoperative morbidity (*p* = 0.457) or mortality (*p* = 0.243) (Table 3).

**Table 3:** Morbidity and mortality data comparison

		PALN +	PALN− [*n* = 111]		*p*-value
		N16+ [*n* = 32]	N+ [*n* = 52]	N0 [*n* = 59]	
Significant morbidity	Yes	10 (31.2)	19 (36.5)	26 (44.1)	0.457
	No	22 (68.8)	33 (63.5)	33 (55.9)	
Re-exploration	Yes	29 (90.6)	47 (90.4)	57 (96.6)	0.367
	No	3 (9.4)	5 (9.6)	2 (3.4)	
Chyle leak	A	4 (12.5)	3 (5.8)	6 (10.2)	0.547
	B	1 (3.1)	5 (9.6)	7 (11.9)	
	C	0 (0.0)	0 (0.0)	0 (0.0)	
	None	27 (84.4)	44 (84.6)	46 (78.0)	
Mortality	No	31 (96.9)	51 (98.1)	54 (91.5)	0.243
	Yes	1 (3.1)	1 (1.9)	5 (8.5)	

*PALN* para-aortic lymph node

Data are expressed as *n* (%)

### Survival Analysis

The median follow-up period was 22 months (17.1–27.3 months). The estimated 3-years OS of the entire cohort of patients was 67% (95% confidence interval [CI] 53–83.5%), with a median DFS of 28.5 months.

#### N16+ versus N16−

**Fig. 2:**
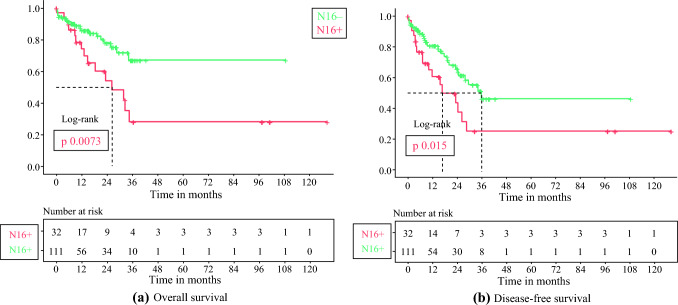
Kaplan–Meier survival curves comparing N16+ versus N16− **a** Overall survival; **b** disease-free survival

The N16− cases had an estimated 3-years OS of 67% (95% CI 53–83.5%), with median survival not reached. In comparison, the N16+ cases had a considerably poor survival, with a median OS of 26.2 months and an estimated 3-years OS of 28% (95% CI 13–60%) [*p* = 0.0073]. Figure 2a shows the OS curves comparing N16+ and N16− cases. The estimated median DFS of N16+ and N16− cases was 16.6 months (3-years DFS of 24.8%) and 35.9 months (3-years DFS of 46%), respectively (*p* = 0.015) (Fig. 2b).

#### N16+ versus N+ and N0

**Fig. 3:**
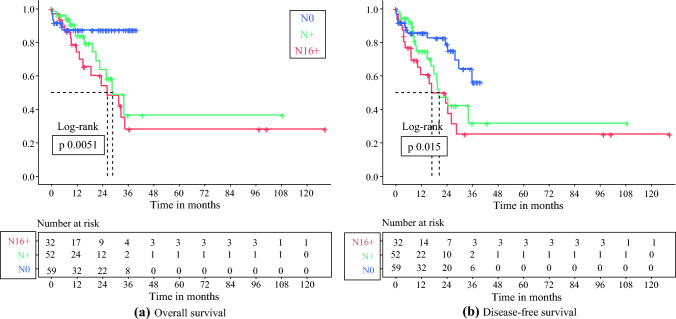
Kaplan–Meier curves comparing survival stratified by nodal involvement: N0, N+ and N16+ subgroups **a** Overall survival; **b** disease-free survival

The median OS for N+ cases was 28.4 months (95% CI 18.9–37.9 months), which was not significantly different from the N16+ group (26.2 months) [*p* = 0.33]. The estimated 3-years OS was 36.5% (95% CI 17–77%). Expectedly, in the N0 subgroup, we noticed a significantly longer survival with an estimated 3-years OS of 87.5% (95% CI 79–96.7%; *p* = 0.0051) (Fig. 3a).

There was no significant difference in the median DFS of the N16+ (16.6 months) and N+ (20.1 months) groups (*p* = 0.24). The N0 subgroup had a longer DFS, with an estimated 3-years DFS of 56.1% (95% CI 38–82.6%; *p* = 0.015) (Fig. 3b).

The median OS of the 10 patients in whom resection was abandoned after a FS analysis showed N16+ was only 9 months, significantly shorter than the N16+ subgroup (*p* = 0.001)

#### N16+ and Factors Predicting Overall Survival

On univariate analysis, the need for vascular resection was associated with adverse outcomes. A higher lymph node ratio (LNR; hazard ratio [HR] 12.2; *p* = 0.021), the presence of PNI on histopathology (*p* = 0.036), the presence of N2 disease (HR 3.86, 95% CI 1.62–9.21; *p* = 0.002) and N16+ (HR 2.433, 95% CI 1.24–4.757; *p* = 0.009) were found to be independent adverse predictors of survival in the study group. Intestinal-type differentiation of adenocarcinoma was found to offer the best prognosis (*p* = 0.028), while the mixed subtype was worse (HR 5.5, 95% CI 1.48–21; *p* = 0.011) (Table 4). On stepwise multivariable Cox regression, an increasing LNR (HR 125, 95% CI 3.52–4464; *p* = 0.008), node-positive status (HR 5.56, 95% CI 1.147– 27.03; *p* = 0.033) and the need for vascular resection (HR 6.803, 95% CI 1.246–37.145, *p* = 0.027) retained prognostic significance.

**Table 4:** Univariate survival analysis of prognostic factors for periampullary cancer

		Overall survival			Disease-free survival		
		Univariate analysis			Univariate analysis		
		HR	95% CI	*p*-value	HR	95% CI	*p*-value
Age, years	<56						
	≥56	1.016	0.985–1.048	0.12	1.016	0.985–1.048	0.317
Sex	Male						
	Female	1.038	0.589–1.829	0.5897	1.206	0.62–2.347	0.581
Clinico-radiological primary site	Ampulla	0.978	0.133–7.216	0.983	0.613	0.148–2.544	0.500
	CBD	0.817	0.091–7.32	0.857	0.587	0.118–2.921	0.515
	Duodenum	0.00	0.00–NR	0.972	0.00	0.00–NR	0.976
Preoperative biliary drainage	ERCP			0.295			0.115
	PTBD	2.937	1.01–8.54	0.048	2.817	1.089–7.284	0.033
	EUS- BD	1.156	0.156–8.56	0.887	1.852	0.444–7.724	0.397
	No	0.672	0.234–1.936	0.462	0.571	0.224–1.458	0.241
Neoadjuvant chemotherapy	Yes						
	No	0.846	0.203–3.53	0.819	1.361	0.330–5.611	0.669
Surgery approach	Open						
	Robotic	0.429	0.059–3.14	0.405	0.580	0.141–2.391	0.451
Vascular resection	Yes						
	No	0.186	0.055–0.628	**0.007**	0.187	0.056–0.621	**0.006**
Histopathology primary site	Duodenum	0.637	0.058–7.042	0.713	0.364	0.038–3.50	0.382
	CBD	1.863	0.338–10.28	0.475	1.769	0.438–7.149	0.424
	Periampullary, NOS	1.244	0.291–5.31	0.768	1.132	0.344–3.729	0.838
	Ampulla	1.672	0.342–8.167	0.526	1.586	0.437–5.757	0.483
Adjuvant treatment	Observation	0.028	0.006–0.132	**0.002**			**0.023**
	CT	4.059	0.0947–17.4	0.059	1.788	0.780–4.098	0.169
	CTRT	24.90	3.92–158	**0.001**	7.09	1.749–28.80	**0.006**
Tumor size		1.098	0.84–1.423	0.478	1.088	0.876–1.350	0.447
LN ratio		12.26	1.46–102	**0.021**	10.9	1.608–74.04	**0.014**
LVI	Present						
	Absent	0.587	0.3–1.14	0.120	0.629	0.356–1.113	0.111
PNI	Present						
	Absent	0.476	0.238–0.953	**0.036**	0.503	0.277–0.914	**0.024**
Margins	Free (R0)						
	Involved (R+)	2.097	0.81–5.41	0.126	1.817	0.772–4.275	0.172
Ampullary subtype	Intestinal			**0.028**			0.218
	Pancreatobiliary	1.038	0.297–3.628	0.953	1.245	0.460–3.37	0.667
	Mixed	5.596	1.48–21.08	**0.011**	3.244	1.001– 0.51	0.050
	Biliary type	4.132	0.423–40.38	0.222	2.412	0.278–20.96	0.425
pT stage	T1			0.129			0.138
	T2	6.588	0.85–50.49	0.070	2.613	0.880–7.756	0.084
	T3	9.936	1.324–74.54	0.026	3.503	1.211–10.13	0.021
	T4	8.58	0.773–95.246	0.080	3.016	0.550–16.55	0.204
pN stage	N0			**0.009**			**0.007**
	N1	2.418	0.897–6.517	0.081	1.5	0.678–3.318	0.317
	N2	3.865	1.620–9.218	**0.002**	2.80	1.44–5.453	**0.002**
N16 involvement	Yes	2.433	1.244–4.757	**0.009**	2.04	1.132–3.675	**0.018**
	No						

Bold values indicate significant *p*-value

*HR* hazard ratio, *CI* confidence interval, *CBD* common bile duct, *ERCP* endoscopic retrograde cholangiopancreatography, *PTBD* percutaneous transhepatic biliary drainage, *CT* chemotherapy, *CTRT* concurrent chemoradiotherapy, *LVI* lymphovascular invasion, *PNI* perineural invasion, *LN* lymph nodes, *NR* not reached, *NOS* not otherwise specified, *EUS-BD* endoscopic ultrasound-guided biliary drainage

#### N16+ and Factors Predicting Disease-Free Survival

Similar to OS, the need for vascular resection was associated with adverse DFS (*p* = 0.006). A higher LNR (HR 10.9; *p* = 0.014), the presence of PNI on histopathology (*p* = 0.024), the presence of N2 disease (HR 2.8, 95% CI 1.44–5.43; *p* = 0.002) and N16+ (HR 2.04, 95% CI 1.132–3.675; *p* = 0.018) were found to be independent adverse predictors of survival in the study group (Table 4). On stepwise multivariable Cox regression, only an increasing LNR (HR 14.37, 95% CI 3.31–62.28; *p* = 0.000) and the need for vascular resection (HR 4.678, 95% CI 1.416–15.45; *p* = 0.011) remained adverse factors.

#### Recurrence Patterns

Among resected patients, recurrence was identified in 40.6% (13/32) of N16+ patients and 18% (20/111) of N16− patients (*p* = 0.003). Recurrence predominantly manifested as distant disease failure (60.6%) in both subgroups (*p* = 0.07), with over half of the patients developing liver metastasis. Retroperitoneal nodal recurrence was more common in N16+ cases, i.e. 30.7% (4/13) as compared with 5% (1/20) in N16− patients (*p* = 0.022).

## Discussion

Lymph node involvement is an important prognostic factor for periampullary cancer. Standard lymphadenectomy for PD includes resection of nodes along the hepatoduodenal ligament (stations 5, 6, 8a, 12b1, 12b2, 12c), the retropancreatic nodes (station 13a, 13b), nodes along the superior mesenteric artery (station 14a, 14b), and anterior surface of the pancreatic head (station 17a, and 17b).^1^ Para-aortic nodal involvement is considered to represent metastatic disease.^1^ The role of radical resection in patients with isolated single-site PALN metastasis remains unclear. Most studies focus on the role of PALN dissection in PDAC. Although these studies report conflicting results, the evidence seems to suggest a benefit in patients who undergo radical resection and complete systemic therapy.^2–9^ Survival after PD for N+ and N16+ is comparable, and in fact significantly improved when compared with palliation alone.^6,25^ Limited published data have evaluated the role of resection with PALN dissection in NPPAC.^26–28^

The current study demonstrated significantly better 3-years OS and DFS for N16− patients compared with the N16+ cases (estimated 3-years OS of 67% vs. 28%; *p* = 0.0073; estimated 3-years DFS 46% vs. 24.8%; *p* = 0.015). However, the median OS, as well as DFS for N16+ cases, was similar to N+ cases (median OS 26.2 vs. 28.4 months, *p* = 0.33; median DFS 20.1 vs. 16.2 months, *p* = 0.24), suggesting that although patients with PALN metastasis (N16+) have poorer outcomes as compared with PALN-negative patients (N16−), the survival in these patients is similar to patients with node-positive (N+) disease.

Nappo et al. reported similar findings in a mixed cohort of 135 PDAC and NPPAC patients, where 15 PALN-positive patients underwent radical resection. OS in PALN-positive (N16+) patients, although significantly poor as compared with node-negative patients (32 months vs. 69 months), was almost similar to node-positive (N+) patients (32 months vs. 34 months).^13^

Similarly, Hempel et al. reported pancreatic resection in 7/67 cases of NPPAC with PALN nodes. They reported similar median OS and progression-free survival (PFS) in patients with PALN-positive (N16+) compared with the PALN-negative group. Furthermore, there was no significant difference in OS as well as PFS between N16+ and node-positive (N+) patients, which corroborates our findings.^15^ Table 5 summarizes the available literature on periampullary cancers and PALN dissection with their outcomes and recommendations.

**Table 5:** Previous studies evaluating the role of PALN dissection in periampullary cancers

	Author	Year	Histology	Total cases	Non-pancreatic periampullary cases with PALN identified	N16+ (%)	Findings	Recommendations
1	**Current study**	**2024**	**Non-pancreatic periampullary cancers**	**887**	**153**	**42 (27.4)**	**3 years OS** **N0: 87.5%** **N+: 36.5%** **N16+: 28%** **Median DFS** **N+: 20.1 months** **N16+: 16.6 months**	**Justify offering radical resection to well-selected patients with isolated PALN metastasis in non-pancreatic periampullary cancers**
2	Bhati et al. ^30^	2023	Pancreatic and non-pancreatic periampullary cancers	114	71	10 (14)	4 years OS N0–N1: 45% N2: 19% N16+: 12% (*p* = 0.006)	In non-pancreatic adenocarcinoma, despite N16+, acceptable long-term survival can be achieved with curative resection
3	Hempel et al. ^15^	2020	Non-pancreatic periampullary cancers	164	67	7 (10)	Median OS PALN−: 29.5 months Median OS of N16+: 24.8 months No significant difference in median OS of the pN1 and N16+ subgroups	Cannot recommend against resection in N16+ cases
4	Bhatti et al. ^11^	2016	Pancreatic and non-pancreatic periampullary cancers	65	40	10 (25)	3 years OS N16+: 60% PALN−: 54%	Curative surgery may benefit and should be considered selectively
5	Nappo et al. ^13^	2015	Periampullary cancers (includes pancreatic head)	135	49	3 (6)	Periampullary mean OS: 32 months Median OS (PDAC): 18 months	Indication for PD
6	Murakami et al. ^14^	2011	Biliary adenocarcinoma	113	63	9 (14)	5 years OS N0: 72% N+: 31% N16+: 24%	Radical surgery should not be abandoned.
7	Connor et al. ^12^	2004	Periampullary cancers (including pancreatic head), distal bile duct	121	11	6 (55)	Lymph node 8a was an independent prognostic factor, but lymph node 16b1 was not	–
8	Yoshida et al. ^10^	2004	Periampullary cancers (including pancreatic head), distal bile duct	101	36	6 (17)	Survival for PALN-negative was significantly better than N16+	In histologically proven N16+, radical surgery was contraindicated

Bold values indicate the findings and details of the current study

*PALN* para-aortic lymph node, *OS* overall survival, *DFS* disease-free survival, *PDAC* pancreatic ductal adenocarcinoma, *PD* pancreaticoduodenectomy, *N0* regional node- and PALN-negative, *N+* regional node-positive, *N16+* PALN-positive

The current study includes 10 patients for whom surgery was abandoned. Multiple station PALN involvement not amenable for R0 resection, patients’ advanced age, comorbidities, and performance status influenced the decision not to offer surgery in these patients. It may not be appropriate to compare the outcomes of these patients with resected patients, as they have more advanced disease or comorbidities and a worse performance status. However, the literature does seem to suggest that patients who undergo resection do better than the patients treated with palliative intent surgical bypass procedures or palliative systemic chemotherapy. Studies have compared PD with extended LN clearance versus palliative surgical bypass with palliative chemotherapy and reported longer OS with PALN+ resections as compared with palliative treatment.^15,29^ In a multicentric cohort study from The Netherlands, the median OS for patients with PALN metastasis who underwent a palliative bypass procedure was 7 months versus 11 months for PD (*p* = 0.049).^29^ However, postoperative morbidity was significantly increased in the resection group (43.8% vs. 7.4%) and multivariate analysis showed that severe comorbidities were independently associated with decreased survival in patients with PALN metastasis.

PD is a complex surgical procedure and is associated with significant morbidity even in specialized high-volume centers.^16,17^ Possible survival benefits of performing radical resections must outweigh the morbidity and mortality risk associated with it. Careful selection to offer resection to patients who are likely to have better perioperative outcomes and complete systemic therapy postoperatively is important. The presence of extensive disease and severe comorbidities may therefore serve as an important criteria in the case of selection. Avoidance of resection in these 10 patients assessed to be ‘high risk’ also likely contributed to keeping perioperative outcomes comparable. In our experience, there was no significant difference in overall morbidity, chyle leak rates, re-exploration rates, and mortality between N16+ and the remainder of the patients, justifying its selective use in patients with good performance status, minimal comorbidities, or low to moderate fistula risk scores.

The incidence of PALN metastasis in periampullary cancers varies from 10.4 to 25% (Table 5).^10–15,29,30^ Overall, the incidence of PALN metastasis in the current study was 4.7% (42/887); however, among patients who underwent PALN sampling or dissection, the incidence of N16 positivity (42/153, 27.4%) was higher than previously reported.^10–15,29,30^ PALN sampling provided an opportunity to make an informed decision to either offer resection or abandon it in 26/86 (30.2%) patients in which FS analysis was performed. Patients who underwent resection could undergo complete clearance. Similarly, among the 67 patients for whom FS analysis of the sampled PALN was not performed, and were only sent for final histology, 16 (23.8%) were found to have PALN metastasis. These findings suggest that PALN sampling and FS analysis may have important implications in deciding the optimal treatment strategy during exploration for PD.

### Strengths and Limitations

The study is retrospective and reports outcomes for a cohort that developed over a period of time. Decisions were case-based and there were no standardized indications for PALN dissection and the use of intraoperative FS analysis in all cases. The study has an inherent selection bias due to its design. We acknowledge that PALN sampling was performed on clinico-radiologic suspicion, which is probably reflected in our relatively higher rates of N16+ patients. Furthermore, as a FS analysis was not performed in all cases, some patients only received para-aortic sampling with PD, likely leading to inadequate staging. These factors limit the generalization of the study findings.

However, to the best of our knowledge, this is the largest experience of resection in isolated N16+ disease in NPPAC. It comprehensively reports outcomes in patients offered PALN dissection in PD for NPPAC. Being a standalone comprehensive cancer center, most treatment decisions were guided by a multidisciplinary tumor board discussion and patients were encouraged to complete their adjuvant therapies as planned.

## Conclusion

In a select group of patients with NPPAC, PD in isolated PALN metastasis was associated with improved OS. Survival in these patients was found to be comparable with regional node-positive patients and was significantly better than palliative treatment alone. Routine intraoperative PALN sampling and its analysis by FS may help determine the optimal strategy for isolated PALN metastasis during surgery for NPPAC.

## Electronic supplementary material

Below is the link to the electronic supplementary material.

Supplementary file 1 (MP4 11500 kb)
